# The efficacy of additional atrial driver ablation in persistent and long-standing persistent atrial fibrillation: a systematic review and meta-analysis of randomized controlled trials

**DOI:** 10.1186/s43044-026-00792-x

**Published:** 2026-11-02

**Authors:** Bernadus Bernardino Bramantyo, Dylan Hanny Firdaus, Juan Aditya, Agnes Vianne, Rizki Hari Mulia, Pirel Aulia Baravia, Ardian Rizal

**Affiliations:** 1 Cardiology Research Office, Department of Cardiology and Vascular Medicine, Dr. Sardjito General Hospital, Yogyakarta, Indonesia; 2 Department of Cardiology and Vascular Medicine, Lawang General Hospital, Malang, Indonesia; 3 Primaya Hospital PGI Cikini, Jakarta, Indonesia; 4 Bachelor Study Program of Medicine, Faculty of Medicine, Universitas Brawijaya, Malang, Indonesia; 5 Amanah Medical Center, Banjarmasin, Indonesia; 6 Department of Cardiology and Vascular Medicine, Faculty of Medicine, Brawijaya University - Saiful Anwar General Hospital, Malang, Indonesia

**Keywords:** Persistent atrial fibrillation, Catheter ablation, Mapping-guided ablation, Atrial driver

## Abstract

**Objective:**

Pulmonary vein isolation (PVI) remains the primary ablation strategy for atrial fibrillation (AF), yet its success in persistent (PeAF) and long-standing persistent AF (LS-PeAF) is suboptimal. Adjunctive ablation targeting atrial drivers has been proposed to enhance outcomes. This systematic review and meta-analysis compared PVI alone with PVI plus atrial driver ablation in PeAF and LS-PeAF.

**Methods:**

Comprehensive searches of PubMed, Scopus, Europe PMC, and Cochrane Library identified randomized controlled trials (RCTs) enrolling adults with PeAF or LS-PeAF undergoing first-time ablation. Eligible studies compared PVI alone versus PVI plus driver ablation and trials utilizing other adjunctive ablations were excluded. Outcomes included recurrence of AF and any atrial tachyarrhythmia (ATA) beyond the blanking period, procedural parameters, and complications. Pooled analyses employed random-effects models to measure risk ratios (RRs) and mean differences (MDs) with 95% confidence intervals (CIs).

**Results:**

Seven RCTs met inclusion criteria. Adjunctive driver ablation modestly improved freedom from AF (RR 1.16; 95% CI, [1.03, 1.31]; *p* = 0.01; I²=61%). However, this finding was accompanied by substantial heterogeneity and was not robust to leave-one-out sensitivity analysis. Driver ablation failed to elevate freedom from any ATA (RR 1.09; 95% CI, [0.98, 1.20]; *p* = 0.11; I²=47%). Procedure duration (MD 37.81 min; 95% CI, [4.42, 71.20]; *p* = 0.03; I²=96%), fluoroscopy time (MD 4.25 min; 95% CI, [2.83, 5.67]; *p* < 0.00001; I²=0%), and ablation time (MD 10.01 min; 95% CI, [0.25, 19.77]; *p* = 0.04; I²=95%) were significantly longer with adjunctive driver ablation. Procedural complication rates were comparable among both groups (RR 1.65; 95% CI, [0.80, 3.43]; *p* = 0.18; I²=20%).

**Conclusion:**

Additional atrial driver ablation modestly improves freedom from AF but does not reduce ATA recurrence, prolonging procedure time without increasing complications.

**Supplementary Information:**

The online version contains supplementary material available at https://doi.org/10.1186/s43044-026-00792-x.

## Introduction

Persistent (PeAF) and long-standing persistent atrial fibrillation (LS-PeAF) represent more advanced subtypes of atrial fibrillation (AF), the predominant sustained disorder affecting cardiac rhythm. Globally, AF was estimated to affect approximately 46.3 million individuals in 2016, with nearly 70% of cases occurring in individuals older than 65 years [1, 2]. Given the ongoing rise in global life expectancy, the global burden of AF is projected to rise nearly two times over next few decades. Longitudinal registry data indicated that over 50% of individuals diagnosed with paroxysmal AF (PAF) progress to PeAF within ten years [3]. Pathophysiologically, the inflammatory response associated with sustained AF promotes fibroblast activation, resulting in the release of growth factors and profibrotic proteins. The consequent atrial fibrosis, in conjunction with ion channel dysfunction, contributes to the maintenance and progression of AF [4]. Compared with PAF, PeAF and LS-PeAF are associated with higher arrhythmic burden, increased complication rates, and greater mortality risk. Adverse clinical outcomes commonly include embolic stroke, heart failure, and cognitive decline such as dementia.

Catheter ablation constitutes the cornerstone of treatment in individuals with AF resistant to antiarrhythmic drugs (AADs). Among various ablation techniques, pulmonary vein isolation (PVI) has emerged as the primary modality of ablation, as the PV region is recognized as the predominant trigger site for AF. Circumferential PVI demonstrates substantial efficacy in PAF, with success rates ranging from 70% to 80%. However, in PeAF and LS-PeAF, catheter ablation outcomes are considerably less favorable [5]. Current evidence indicates that PVI alone achieves only a 21% to 43% success rate in this population [6, 7], reflecting the multifactorial mechanisms sustaining AF in its persistent forms. These include PV reconnection and the presence of additional atrial drivers or non-PV foci, which perpetuate arrhythmogenesis. Consequently, there is growing interest in developing adjunctive ablation strategies to improve outcomes in PeAF and LS-PeAF. It has been hypothesized that targeting atrial drivers in addition to PVI may enhance procedural efficacy. Nonetheless, existing studies have reported inconsistent results regarding atrial tachycardia (ATA) recurrence following such interventions [8–11]. Given this background, the current investigation aims to assess the clinical effectiveness of adjunctive atrial driver ablation among individuals diagnosed with PeAF and LS-PeAF undergoing PVI.

## Methods

### Study protocol

This study was performed based on the updated guideline of the Preferred Reporting Items for Systematic reviews and Meta-Analyses (PRISMA) 2020 statement [12]. Previously, the protocol of this study has been registered in the International Prospective Register of Systematic Reviews (PROSPERO) database with the study number of CRD420251150110.

### Search strategy

A systematic database search was carried out by six investigators (BBB, DHF, JA, AV, RHM, and PAB) across numerous databases, namely PubMed, Scopus, Europe PMC, and Cochrane Library. The objective was to identify relevant literature evaluating the comparative outcomes of PVI alone versus PVI combined with adjunctive atrial driver ablation in patients with PeAF and/or LS-PeAF. Eligible publications were considered from database inception through July 2026. The literature search utilized a structured combination of free-text terms to obtain a thorough representation of the relevant literature: ((((“atrial fibrillation”) OR (“AF”)) AND ((“persistent atrial fibrillation”) OR (“long-standing persistent atrial fibrillation”) OR (“longstanding persistent atrial fibrillation”))) AND ((“catheter ablation”) OR (“atrial driver ablation”) OR (“rotor ablation”) OR (“focal impulse and rotor modulation”) OR (“FIRM”) OR (“driver ablation”) OR (“driver mapping”) OR (“mapping-guided ablation”) OR (“computational mapping”))) AND ((“randomized controlled trial”) OR (“randomized”) OR (“randomised”) OR (“RCT”)). The detailed search strategy is provided in Supplementary Table 1.

### Eligibility criteria

Predefined eligibility criteria were established prior to the literature search. Studies were deemed eligible when following criteria were fulfilled: [1] randomized controlled trials (RCTs); [2] the study enrolled patients aged ≥ 18 years with PeAF and/or LS-PeAF; [3] the study compared PVI alone with PVI plus adjunctive atrial driver ablation; [4] the study reported one of the following outcomes—namely (a) AF recurrence after the blanking period, (b) any atrial tachyarrhythmia (ATA) recurrence after the blanking period (including AF, atrial tachycardia, or atrial flutter), (c) procedural duration, (d) fluoroscopy time, (e) radiofrequency application time, or (f) procedural complications; and [5] the study was written and published in English. No restrictions were imposed on the mapping techniques used to identify atrial drivers for adjunctive ablation. Exclusion criteria encompassed: [1] observational studies, review articles, case reports, editorials, book chapters, letters, or guidelines; [2] studies enrolling patients with arrhythmias other than PeAF or LS-PeAF; [3] studies conducting atrial substrate ablation; [4] studies performing additional ablation strategies beyond PVI and/or atrial driver ablation or stepwise ablation approach; [5] single-arm studies evaluating only one intervention; and [6] articles not published in English.

### Study selection and data extraction

The identified records were imported into Rayyan website for study selection. Duplicate records were excluded, after which three independent reviewers (BBB, DHF, and PAB) screened the relevance of titles and abstracts. Potentially eligible studies were retrieved and evaluated for compliance with the inclusion criteria. The overall workflow of study selection was verified by three independent reviewers (AV, RHM, and DHF). Data extraction was performed using Google Sheet. Extracted variables included study ID (first author’s last name and publication year), study center, design, sample size for each intervention arm, mean age, number of LS-PeAF cases, comorbidities, CHA₂DS₂-VASc score, mapping approach, and prespecified outcomes of interest. Numerical data were presented as mean values with their corresponding standard deviations (SD). In instances where data were reported as medians, the median was treated as the mean, and the interquartile range (IQR) was converted to an SD by dividing it by 1.35. For studies with incomplete data, the corresponding authors were contacted; studies lacking author response were subsequently excluded. Discrepancies during this process were discussed until a consensus was achieved under the supervision of the senior author (AR).

### Definitions

PeAF is defined as AF episodes persisting for more than seven days, including those requiring pharmacological or electrical cardioversion for termination [13]. LS-PeAF, in contrast, refers to AF sustained beyond 12 months. Atrial drivers represent mappable electrophysiological mechanisms that sustain fibrillatory conduction. These drivers can be identified using various advanced mapping techniques, including dominant frequency analysis, focal impulse and rotor modulation (FIRM) assessment, and non-contact body surface mapping [14]. Catheter ablation was undertaken with radiofrequency energy which produces controlled thermal injury to achieve tissue necrosis. A post-ablation blanking period—typically the initial three months following the procedure—was designated as an interval during which the recurrence of ATAs is not regarded as procedural failure. Recurrence beyond the blanking period was considered as a documented occurrence of AF or ATA lasting at least 30 s. Procedural complications were defined as any adverse event related to the intervention occurring intra-procedurally or within 30 days post-ablation. In the present study, procedural complications included pericardial effusion or cardiac tamponade, pericarditis, stroke or transient ischemic attack (TIA), hemoptysis, pleural effusion, pneumonia, and vascular access-related complications.

### Risk of bias assessment

The methodological rigor of RCTs was evaluated utilizing the Cochrane Risk of Bias 2 (RoB 2) tool which evaluates six domains. A study was deemed low risk when all domains were evaluated to be of low risk. When one domain raised concerns, the trial was categorized as having some concerns. A high risk of bias was determined when at least one domain was rated as high risk. Furthermore, studies in which the majority of domains indicated some concerns were also considered to have an overall high risk of bias [15].

### Statistical analysis

Statistical analyses were conducted by employing Review Manager (RevMan) version 5.4. Pooled effect sizes for dichotomous and continuous outcomes were estimated with a random-effects model (DerSimonian–Laird), regardless of the value of the heterogeneity. Dichotomous and continuous outcomes were presented as risk ratios (RRs) and mean differences (MDs), each with corresponding 95% confidence intervals (CIs). An exploratory subgroup analysis was conducted according to the atrial driver ablation strategy. Heterogeneity of the statistical model was quantified using the I² statistic, with thresholds of < 25%, 25–50%, 50–75%, and > 75% representing no, low, moderate, and high heterogeneity, respectively. Statistical significance was defined as *p* < 0.05. The statistical model is represented using forest plots. Sensitivity analysis was performed through a leave-one-out analysis in Stata 18 (StataCorp, College Station, TX). This approach involves sequentially excluding individual study at a time to assess the impact of each study on the overall results. The statistical model was considered robust when the p-values remained consistent with the primary analysis across all stages of the sensitivity analyses. Egger’s test and Begg’s test were analyzed to determine publication bias, conducted in Stata 18, with *p* < 0.05 indicating relevant publication bias.

## Results

### Characteristics of included studies

**Fig. 1:**
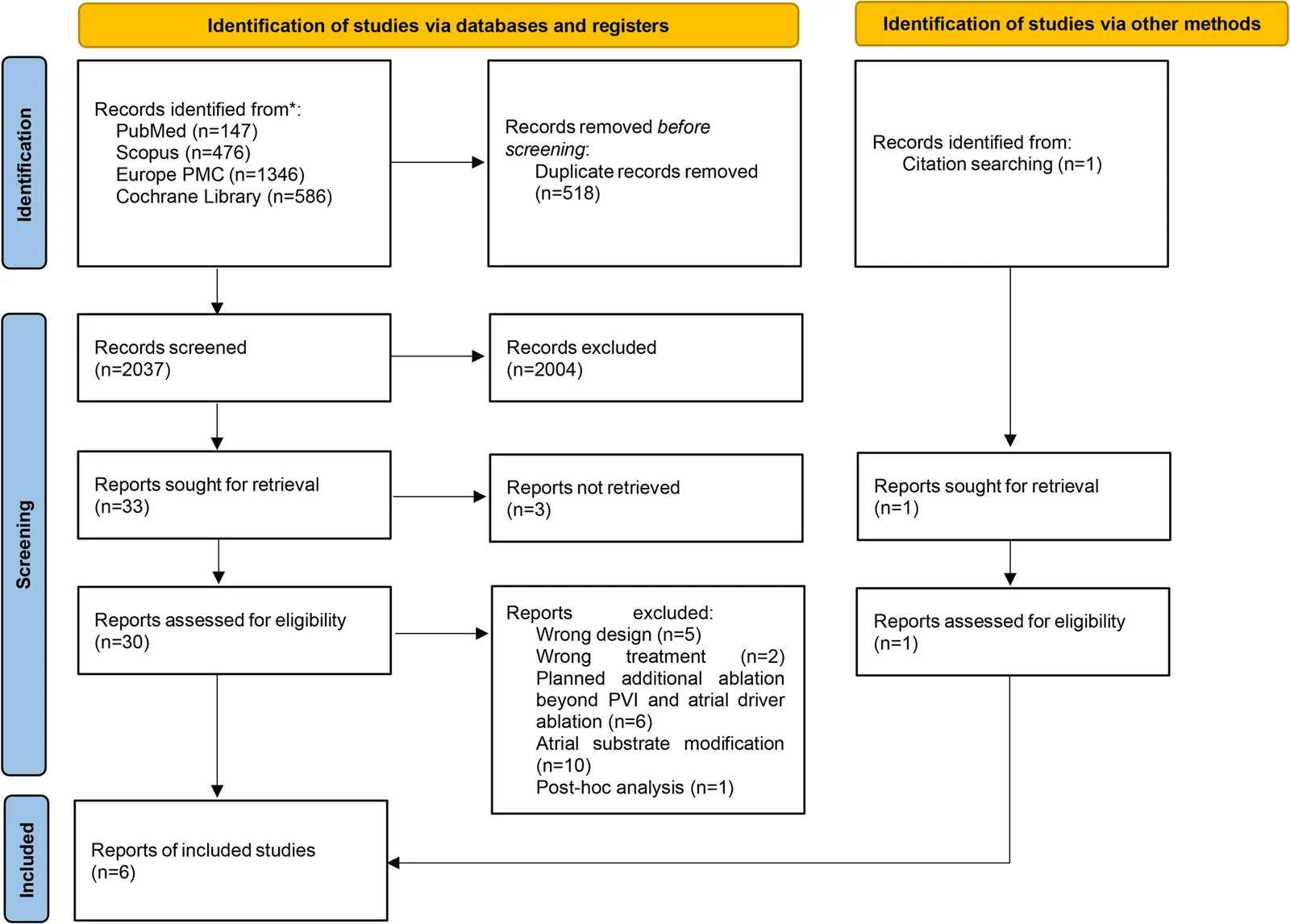
PRISMA diagram representing study selection process

A total of 2555 records were identified through database searches and additional sources. After removing 518 duplicate records, 2004 records were excluded following title and abstract screening. Thirty full-text articles were subsequently excluded for reasons detailed in the PRISMA flow diagram. One additional eligible study was identified through citation tracking, giving rise to a final inclusion of 7 studies in the quantitative synthesis. The study selection flow is illustrated in Fig. 1.

Overall, the included trials enrolled 1395 patients (PVI+driver ablation = 696, PVI only = 699) across both multicenter and single-center studies conducted in Germany, Belgium, the Netherlands, France, Spain, Japan, China, the United States, Canada, and South Korea. However, the quantitative analysis included only 1359 patients (683 in the PVI + driver ablation group and 676 in the PVI-only group) due to loss to follow-up in studies by Atienza et al. (2014), Brachmann et al. (2025), and Deisenhofer et al. (2025) [10, 16, 17]. These studies evaluated the comparative effectiveness of PVI alone versus PVI combined with adjunctive atrial driver ablation in patients with PeAF or LS-PeAF. Three studies identified atrial drivers by mapping regions of high dominant frequency within the left atrium [10, 18, 19]. Baek et al. further implemented patient-specific computational modeling of atrial fibrillation and performed dominant frequency analysis using CUVIA version 2.5 [18]. Brachmann et al. employed FIRM, whereby arrhythmic sources were identified through continuous ≥ 1-minute electrogram recordings from both atria using a 64-pole FIRMap catheter [16]. Deisenhofer et al. characterized atrial drivers based on spatiotemporal dispersion patterns derived from multipolar intracardiac electrograms analyzed with an artificial intelligence–based tool (Volta AF-Xplorer) [17]. Similarly, Hu et al. identified atrial drivers by detecting bipolar electrograms exhibiting spatial dispersion [20]. Takahashi et al. performed atrial driver ablation by targeting regions demonstrating focal activation patterns during atrial fibrillation, as identified from 30-second electrogram recordings obtained with a PentaRay catheter [8]. The key characteristics of the included studies are summarized in Table 1.

**Table 1:** Baseline characteristics of the included studies

Study ID	Center	Intervention Arm	Age (mean ± SD)	Sample Size	Male (*n*, %)	LS-PeAF (*n*, %)	AF Duration (months) (mean ± SD)	CHA_2_DS_2_-VASc (mean ± SD)	LVEF (%) (mean ± SD)	Comorbidities (*n*, %)	Atrial Driver Detection	Follow-Up Duration (months)
Atienza 2014	Spain	PVI Only	54 ± 10	58	47/58 (81.0)	N/A	15.84 ± 13.08	N/A	60 ± 3.9	HT = 20 (34.5) DM = 5 (8.6) Dyslipidemia = 20 (34.5) Alcohol = 2 (3.4) Smoking = 22 (37.9)	Dominant frequency	12
		PVI + Atrial Driver Ablation	55 *±* 9	59	48/59 (81.4)		18 ± 9.6		60 ± 5.2	HT = 29 (49.2) DM = 8 (13.6) Dyslipidemia = 15 (25.4) Alcohol = 4 (6.8) Smoking = 26 (44.1)		
Baek 2021	South Korea	PVI Only	60.2 ± 11.1	83	53/83 (63.9)	54/83 (65.1)	43.9 ± 44.5	2.1 ± 1.6	60.1 ± 9.0	HT = 42 (50.6) DM = 20 (24.1) HF = 18 (21.7) Stroke = 14 (16.9) Vascular Disease = 4 (4.8)	Dominant frequency	16.3 ± 5.3
		PVI + Atrial Driver Ablation	58.3 ± 11.5	87	67/87 (77.0)	52/87 (59.8)	36.7 ± 38.8	2.1 ± 1.5	60.0 ± 7.6	HT = 51 (58.6) DM = 19 (21.8) HF = 22 (25.3) Stroke = 13 (14.9) Vascular Disease = 9 (10.3)		
Brachmann 2025	Germany	PVI Only	62.7 ± 8.7	179	127/179 (70.9)	N/A	N/A	N/A	54.5 ± 8.9	N/A	FIRM	12
		PVI + Atrial Driver Ablation	64.2 ± 8.4	171	126/171 (73.7)				53.8 ± 8.0			
Deisenhofer 2025	US, France, Germany, Belgium, Netherlands	PVI Only	64.9 ± 8.5	183	148/183 (80.9)	27/183 (14.8)	28.8 ± 39.6	2.0 ± 1.4	54.3 ± 9.6	HT = 109 (59.6) DM = 29 (15.8) Stroke = 12 (6.6) Vascular Disease = 28 (15.3) Dyslipidemia = 58 (31.7) Obesity = 78 (42.6) Sleep Apnea = 44 (24.0)	Spatio-temporal dispersion	12
		PVI + Atrial Driver Ablation	66.4 ± 8.5	187	145/187 (77.5)	40/187 (21.4)	32.4 ± 44.4	2.3 ± 1.4	55.3 ± 9.3	HT = 118 (63.1) DM = 33 (17.6) Stroke = 15 (8.0) Vascular Disease = 27 (14.4) Dyslipidemia = 62 (33.2) Obesity = 78 (41.7) Sleep Apnea = 39 (20.9)		
Hu 2021	China	PVI Only	64.9 ± 10.5	62	25/62 (40.3)	8/62 (12.9)	8.1 ± 4.5	2.2 ± 1.5	61.8 ± 4.6	HT = 32 (51.6) DM = 12 (19.4) HF = 1 (1.6) Stroke = 5 (8.1) CAD = 11 (17.7)	Bipolar electrogram dispersion	16.9 ± 6.5
		PVI + Atrial Driver Ablation	64.6 ± 7.7	62	22/62 (35.5)	8/62 (12.9)	7.4 ± 4.6	2.3 ± 1.2	61.6 ± 3.9	HT = 30 (48.4) DM = 12 (19.4) HF = 2 (3.2) Stroke = 4 (6.5) CAD = 7 (11.3)		
Kumagai 2026	Japan	PVI Only	65 ± 9.1	32	30/32 (93.8)	17 (53.1)	12 ± 19.3	N/A	60 ± 6.7	HT = 17 (53.1) DM = 4 (12.5) HF = 3 (9.4) Stroke = 1 (3.1)	Dominant frequency	12
		PVI + Atrial Driver Ablation	62 ± 8.5	32	25/32 (78.1)	12 (37.5)	9 ± 11.9		61 ± 7.8	HT = 19 (58.4) DM = 6 (18.8) HF = 3 (9.4) Stroke = 0 (0)		
Takahashi 2024	Japan	PVI Only	66.8 ± 8.3	102	84/102 (82.7)	18/102 (17.7)	5.6 ± 4.1	2 ± 1.5	59.1 ± 8.4	HT = 35 (34.3) DM = 19 (18.6) HF = 9 (8.8)	Focal activation sites	24
		PVI + Atrial Driver Ablation	66.4 ± 8.2	98	81/98 (82.4)	20/98 (20.4)	5.5 ± 3.9	2 ± 1.5	61.8 ± 8.7	HT = 30 (30.6) DM = 20 (20.4) HF = 11 (11.2)		

SD: standard deviation

AF: atrial fibrillation; DM: diabetes mellitus; FIRM: focal impulse and rotor modulation; HF: heart failure; HT: hypertension; LS-PeAF: long-standing persistent atrial fibrillation; LVEF: left ventricular ejection fraction; PVI: pulmonary vein isolation

### Risk of bias

**Fig. 2:**
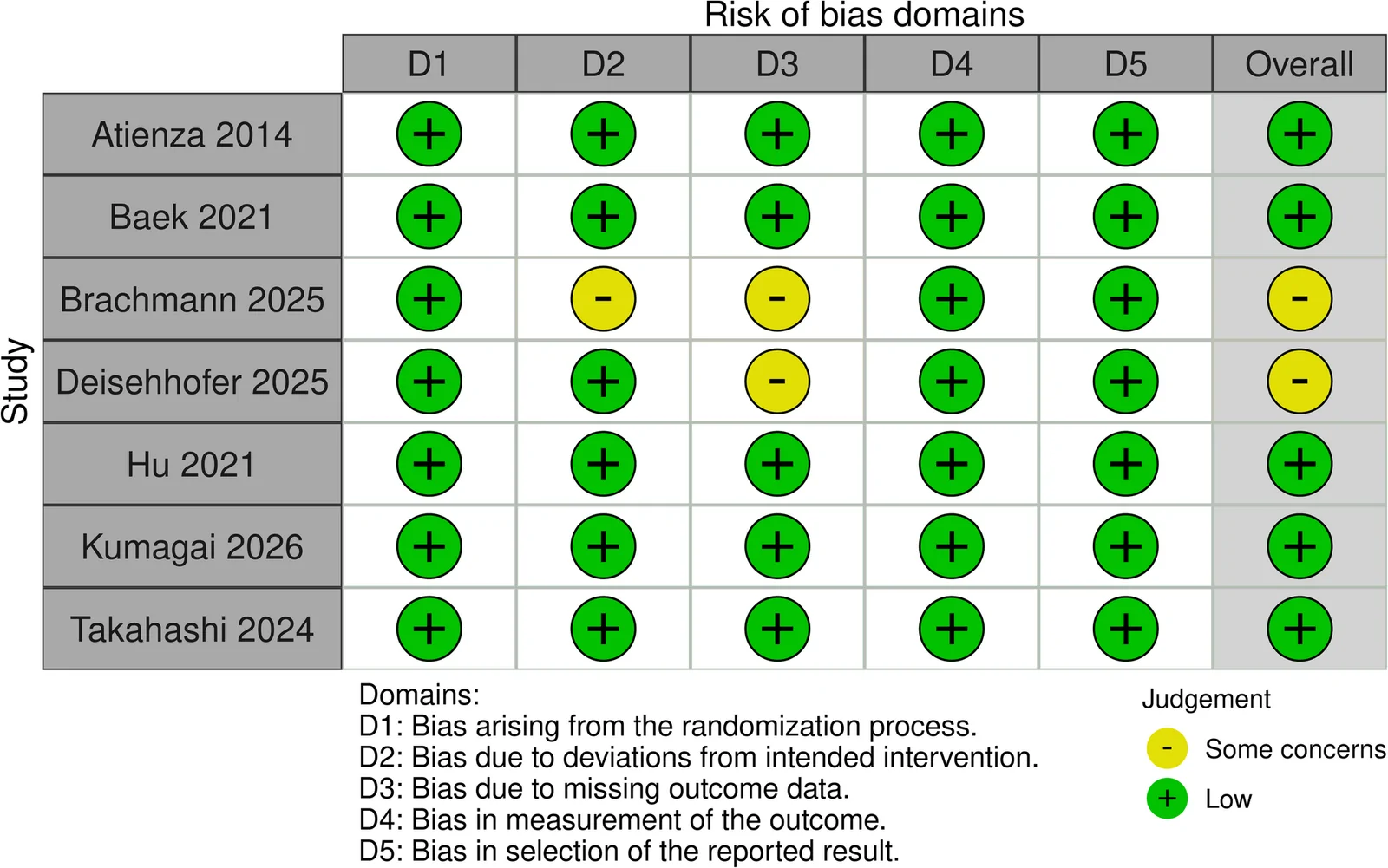
The risk of bias of the included studies

Five studies were judged as having low risk of biases, suggesting that their methodological rigor and reporting quality were generally sound and unlikely to pose substantial threats to internal validity. Two studies were judged to raise some concerns, reflecting potential methodological limitations that may reduce confidence in its findings, although these concerns did not warrant classification as high risk of bias. The identified issues were primarily related to deviations from the intended interventions and incomplete or insufficiently detailed reporting of prespecified outcomes, which limited comprehensive appraisal of the study results. Particularly, Brachmann et al. (2025) and Deisenhofer et al. (2025) reported loss to follow-up among substantial number of enrolled patients. Furthermore, a substantial proportion of patients in the study by Brachmann et al. (2025) underwent additional ablation procedures. A graphical summary of the RoB 2 assessment is shown in Fig. 2.

### Meta-analysis

**Fig. 3:**
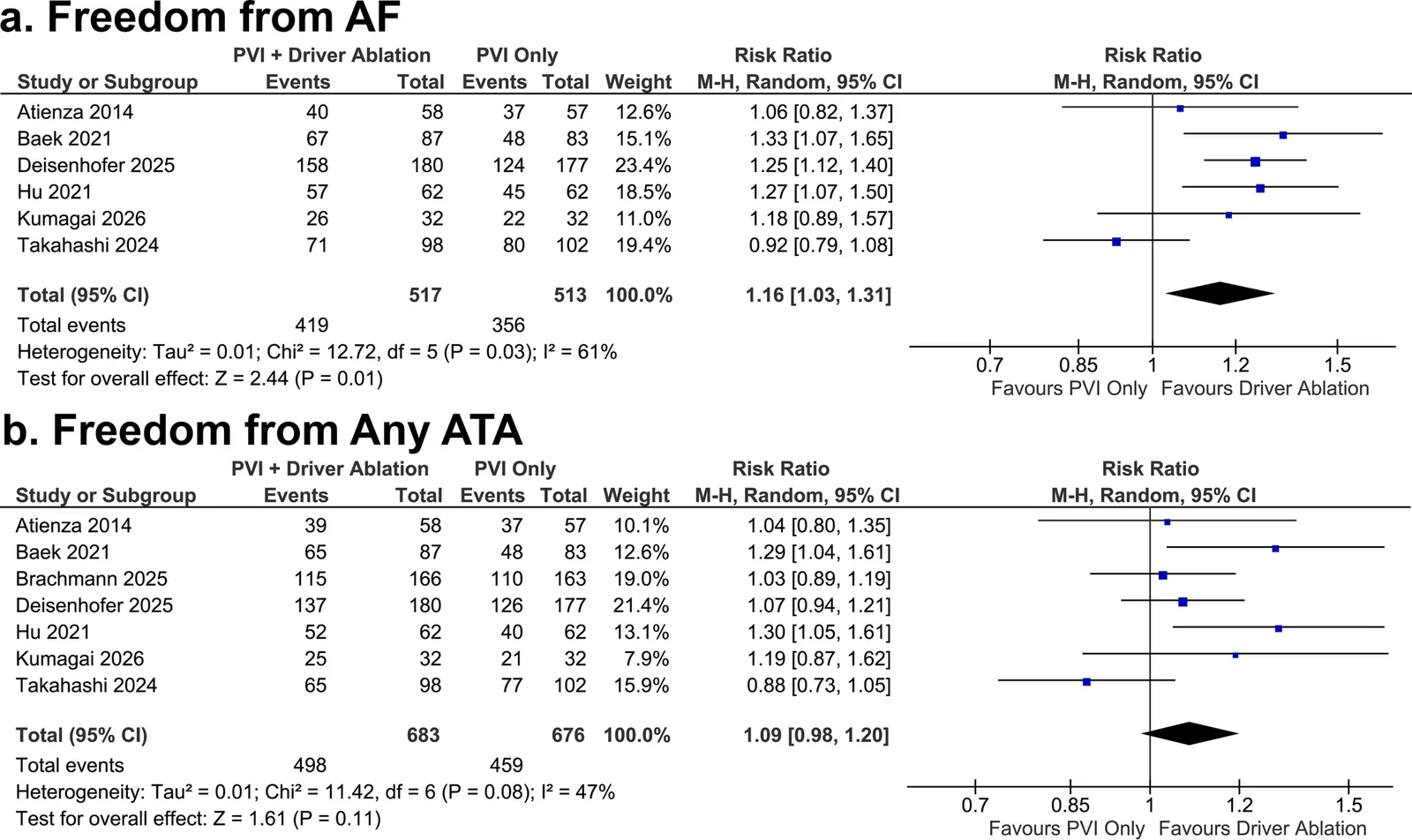
Forest plots for efficacy outcomes. (**a**) Freedom from AF. (**b**) Freedom from any ATA

Meta-analysis revealed a modestly enhanced likelihood of freedom from AF after the blanking period with PVI plus atrial driver ablation compared PVI alone (RR 1.16; 95% CI, [1.03, 1.31]; *p* = 0.01; I²=61%; Fig. 3a). In contrast, adjunctive atrial driver ablation was not associated with significant difference in terms of freedom from any atrial tachyarrhythmia between both groups (RR 1.09; 95% CI, [0.98, 1.20]; *p* = 0.11; I²=47%; Fig. 3b). Exploratory subgroup analyses based on atrial driver ablation strategy revealed that only dominant frequency-, spatiotemporal dispersion- and bipolar electrogram dispersion-guided approaches were associated with increased freedom from AF. For recurrence of any ATA, a significant benefit was observed with dominant frequency- and bipolar electrogram dispersion-guided ablation. Given the limited number of available studies and the resulting limited statistical power, trends observed in the subgroup analyses should not be interpreted as establishing the superiority of any catheter ablation method. The complete subgroup analysis results are shown in Supplementary Figs. 1.

**Fig. 4:**
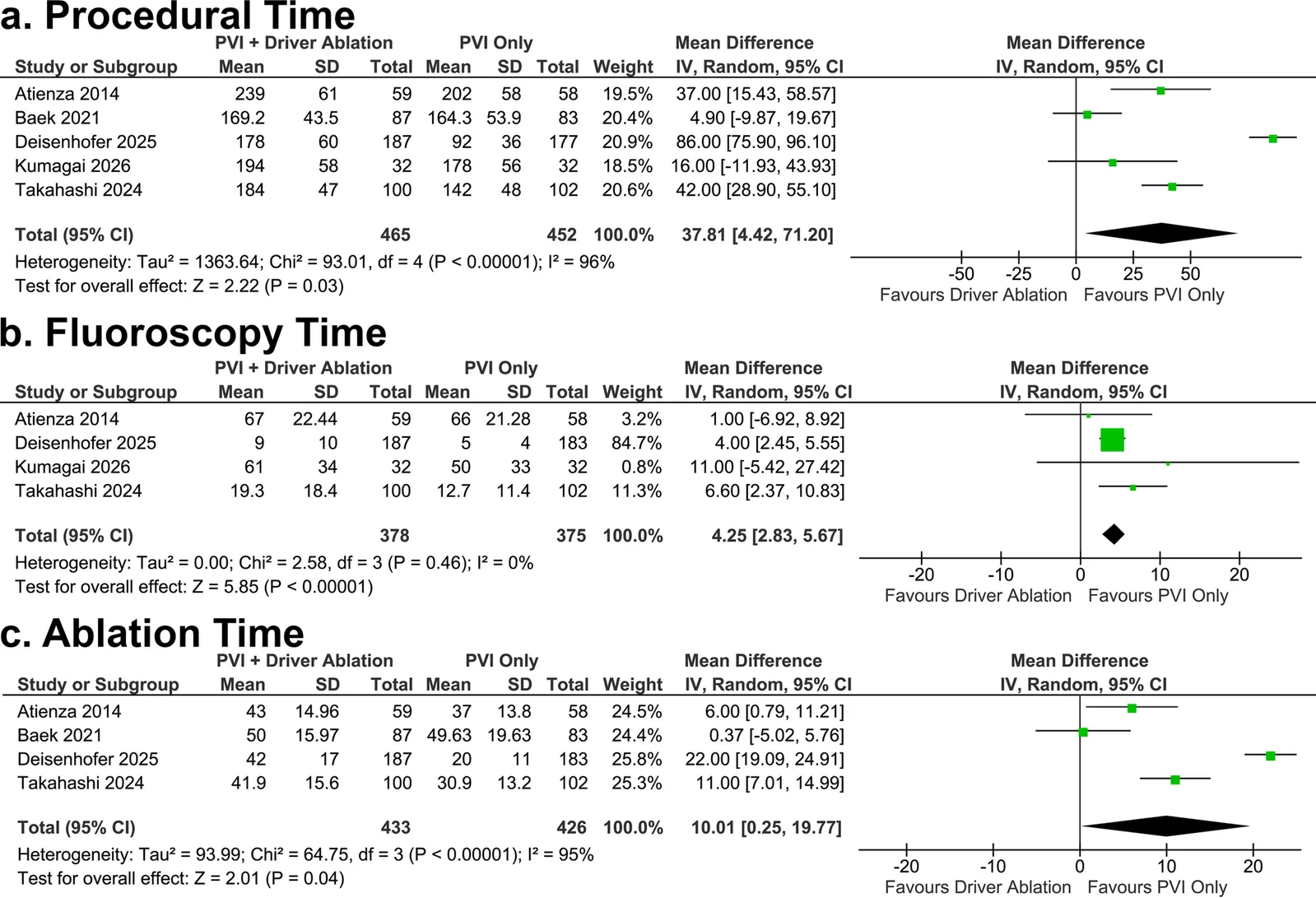
Forest plots for all procedural outcomes. (**a**) Procedural time. (**b**) Fluoroscopy time. (**c**) Ablation time

**Fig. 5:**
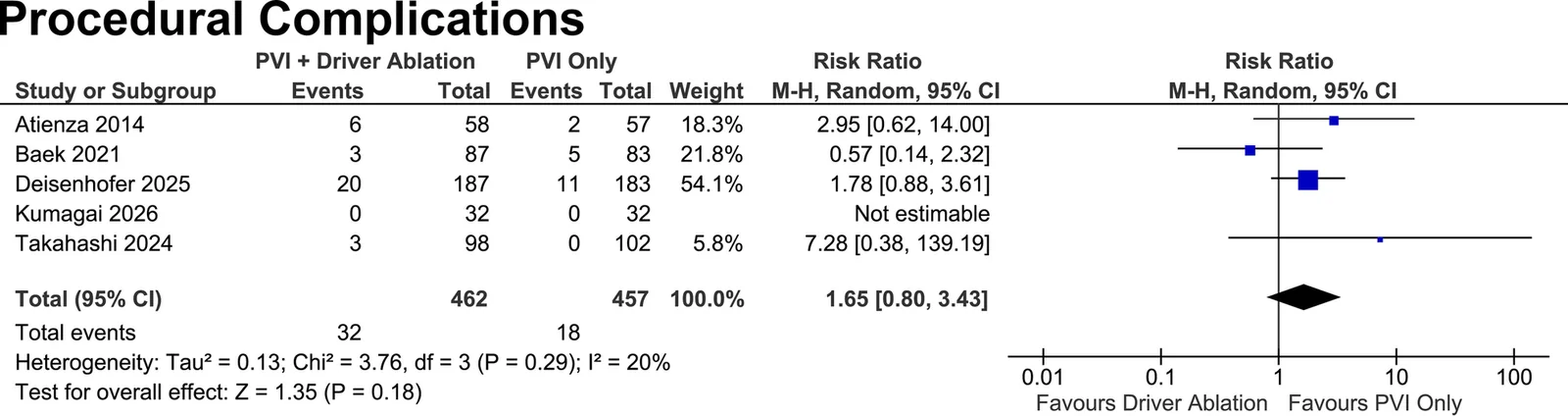
Forest plot for safety outcome of procedural complications

Regarding procedural characteristics, pooled analysis demonstrated that procedural time was significantly longer in the PVI plus atrial driver group (MD 37.81 min; 95% CI, [4.42, 71.20]; *p* = 0.03; I²=96%; Fig. 4a), with additional increases in fluoroscopy time (MD 4.25 min; 95% CI, [2.83, 5.67]; *p* < 0.00001; I²=0%; Fig. 4b) and ablation time (MD 10.01 min; 95% CI, [0.25, 19.77]; *p* = 0.04; I²=95%; Fig. 4c). Safety outcome analysis showed insignificant difference in the overall risk of procedural complications between the two strategies (RR 1.65; 95% CI, [0.80, 3.43]; *p* = 0.18; I²=20%; Fig. 5). Notably, the study parameters were inconsistently reported across the included studies, resulting in varying numbers of studies available for each outcome, as detailed in Table 2. This limitation may have reduced the statistical power of the corresponding models. These findings are summarized in Table 2.

**Table 2:** Summary of study findings

Outcomes	Number of Studies	PVI+Atrial Driver Ablation		PVI Alone		Model	RR	95% CI	*P*-Value of Heterogeneity	*P*-Value of Egger’s Test	*P*-Value of Begg’s Test	*P*-Value
		Event, *n*	Total, *n*	Event, *n*	Total, *n*							
Freedom from AF	6	419	517	356	513	Random	1.16	1.01 to 1.31	0.03	0.9377	0.7071	0.01
Freedom from Any ATA	7	498	683	459	676	Random	1.09	0.98 to 1.20	0.08	0.3408	0.7639	0.11
Procedural Complications	5	32	462	18	457	Random	1.65	0.80 to 3.43	0.29	0.7659	0.8065	0.18
**Outcomes**	**Number of Studies**	**PVI+Atrial Driver Ablation (Total**,** n)**		**PVI Alone (Total**,** n)**		**Model**	**MD**	**95% CI**	**P-Value of Heterogeneity**	**P-Value of Egger’s Test**	**P-Value of Begg’s Test**	**P-Value**
Procedural Time	5	465		452		Random	37.81	4.42 to 71.20	< 0.00001	0.2835	0.4624	0.03
Fluoroscopy Time	4	378		375		Random	4.25	2.83 to 5.67	0.46	0.6179	1.0000	< 0.00001
Ablation Time	4	433		426		Random	10.01	0.25 to 19.77	< 0.00001	0.0000	0.0894	0.04

CI: confidence interval; MD: mean difference; RR: risk ratio

AF: atrial fibrillation; ATA: atrial tachycardia; PVI: pulmonary vein isolation

### Heterogeneity and publication bias

Substantial heterogeneity was identified across most evaluated outcomes. For efficacy endpoints, the I² statistics for freedom from AF exceeded 50%, with corresponding p-values below 0.05. Similar findings were noted for procedural outcomes—procedural duration, fluoroscopy time, and ablation time—all of which demonstrated elevated I² values. In contrast, the safety endpoint of procedural complications showed low heterogeneity, with non-significant statistical findings. Assessment of publication bias revealed no statistical evidence of relevant publication bias, as indicated by Egger’s and Begg’s test results (*p* > 0.05), with the exception of ablation time. Considering the limited number of included studies, this observation should be interpreted as exploratory. Table 2 provides a summary of heterogeneity and publication bias across all endpoints.

### Sensitivity analysis

**Fig. 6:**
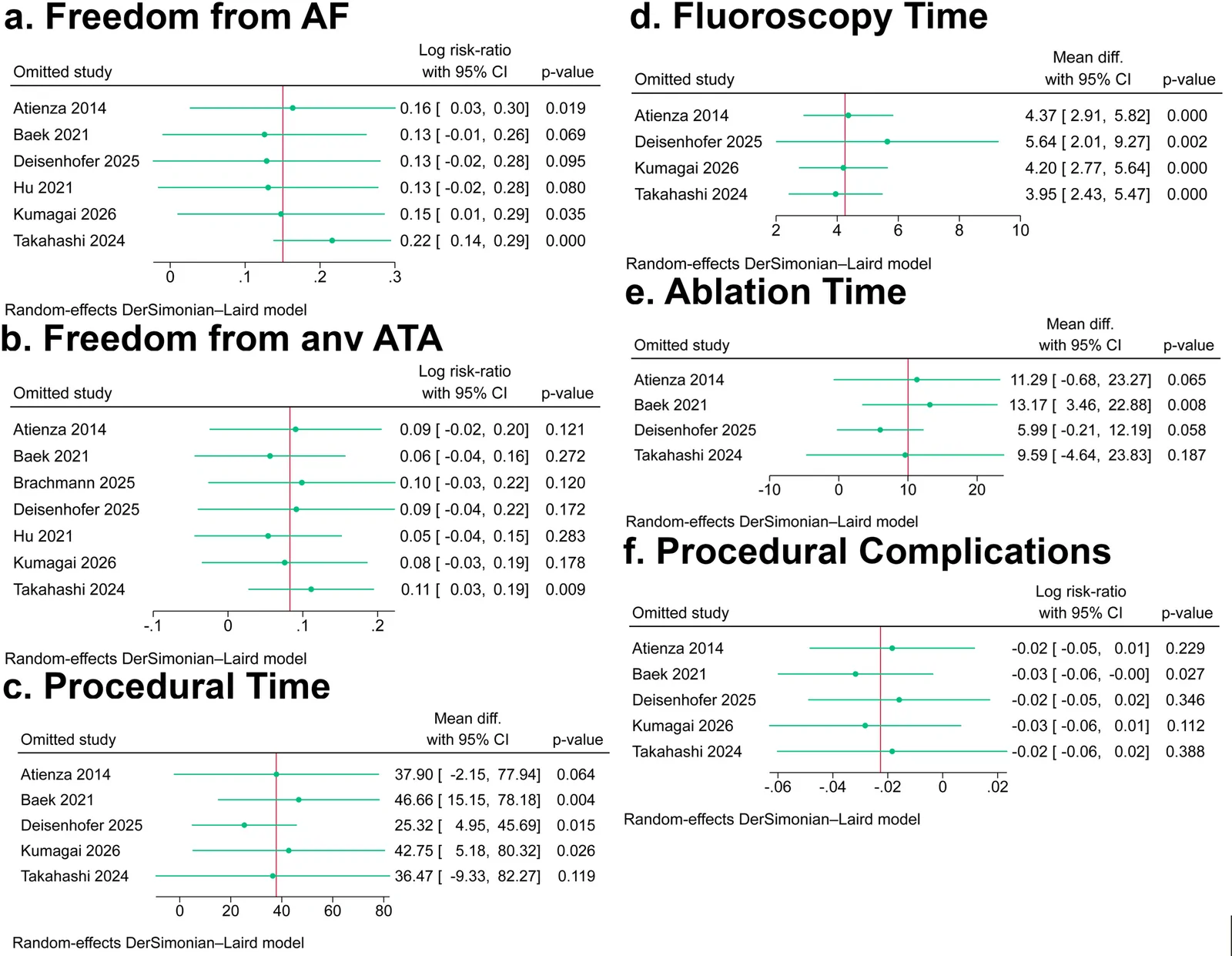
Forest plots for the sensitivity analyses. (**a**) Freedom from AF. (**b**) Freedom from any ATA. (**c**) Procedural time. (**d**) Fluoroscopy time. (**e**) Ablation time. (**f**) Procedural complications

Sensitivity analyses revealed that the findings across all outcomes were not fully robust to the sequential exclusion of individual studies. Although additional atrial driver ablation was linked with a significantly greater likelihood of freedom from AF, this finding was not robust across sensitivity analyses and this result should be interpreted cautiously. Similar patterns were observed for procedural time and ablation time. A robust statistical model was supported only for the analysis of fluoroscopy time. Conversely, for outcomes that did not reach statistical significance in the primary analysis, the exclusion of a single study in each case resulted in statistically significant findings, suggesting that these studies exerted a disproportionate influence on the corresponding statistical models. In addition, half of the clinical endpoints exhibited substantial heterogeneity, as depicted in Table 2. The results of the sensitivity analyses are presented as forest plots in Fig. 6.

## Discussion

In this meta-analysis, we evaluated the clinical efficacy, procedural characteristics, and safety profile of adjunctive atrial driver ablation performed in combination with conventional PVI in patients with PeAF and LS-PeAF. This study examined an AF ablation approach centered on atrial driver mapping, emphasizing a comprehensive mapping-guided strategy rather than the assessment of an individual electrophysiological mechanism. The pooled analysis demonstrated that the addition of atrial driver ablation conferred a modest increase in freedom from AF compared with PVI alone (RR 1.16; 95% CI, [1.03, 1.31]; *p* = 0.01). In contrast, the improvement in freedom from ATA did not achieve statistical significance (RR 1.09; 95% CI, [0.98, 1.20]; *p* = 0.11). These efficacy benefits were accompanied by procedural trade-offs, including longer total procedure duration, increased fluoroscopy exposure, and extended ablation time. Although the combined strategy was associated with a numerically higher incidence of procedural complications, this difference was not statistically significant (RR 1.65; 95% CI, [0.80, 3.43]; *p* = 0.18).

Pulmonary veins and their antral regions have long been recognized as the predominant sources of ectopic triggers initiating AF, particularly in patients with paroxysmal AF. However, growing evidence indicates that non–PV foci can act as critical atrial drivers sustaining AF, especially in patients with non-paroxysmal forms of the arrhythmia. These extrapulmonary sources are commonly identified within the atrial roof, left atrial appendage, interatrial septum, and coronary sinus [21, 22]. Lim et al. demonstrated that patients with longer AF duration and greater arrhythmic complexity exhibit a higher burden of atrial drivers originating outside the PVs [23]. Furthermore, histopathological and electrophysiological studies of human atrial tissue have suggested that AF maintenance may involve localized intramural microanatomic reentrant circuits, which are often undetectable using conventional single-surface mapping techniques [24]. In response to these limitations, advanced mapping technologies—including electrogram-guided mapping, phase mapping, and noninvasive body surface mapping—have been developed to enhance the identification of arrhythmogenic substrates and guide ablation strategies [22, 25, 26]. These approaches aim to enable patient-specific, substrate-guided ablation, thereby improving procedural efficacy and reducing recurrence of atrial tachyarrhythmias, including AF. Nevertheless, the precise mechanisms underlying AF perpetuation remain incompletely elucidated, contributing to variability in ablation outcomes and continued challenges in achieving durable arrhythmia control [4].

Consistent with this evolving paradigm, our meta-analysis demonstrated a modest improvement in freedom from AF with PVI combined with additional ablation (RR 1.16; 95% CI, [1.03, 1.31]; *p* = 0.01). This finding supports the concept that personalized ablation strategies, rather than PVI alone, may confer incremental clinical benefit in patients with non-paroxysmal AF, despite heterogeneity in methods used to identify atrial drivers. In the study by Baek et al., the use of CUVIA version 2.5 to identify dominant frequency (DF) regions enabled tailored driver ablation, which proved superior to ablation targeting complex fractionated atrial electrograms (CFAEs) or phase singularities in PeAF [18]. Deisenhofer et al. further incorporated artificial intelligence–based approaches to objectively map spatiotemporal dispersion during AF ablation, albeit at the expense of longer procedural and ablation durations. Notably, this individualized ablation strategy appeared to provide greater benefit in patients with prolonged PeAF (AF duration > 6 months) and more complex atrial driver distribution outside the PVs. Driver-targeted ablation may also be particularly advantageous in obese patients, in whom adverse atrial remodeling predisposes to AF persistence and suboptimal outcomes following conventional ablation. In this context, integration of high-density mapping with driver ablation may yield superior clinical outcomes in selected patient subgroups [20].

Despite these findings, substantial heterogeneity was observed among the included studies (I²=61%). This was further limited by the lack of robustness observed in the leave-one-out sensitivity analysis. This finding was largely driven by variability in the methodologies used for atrial driver mapping. Additional studies assessing specific atrial driver mapping approaches are warranted to better elucidate the performance characteristics of each method. In contrast to the pooled results, Takahashi et al. reported no additional benefit of adjunctive focal activation site ablation in reducing AF recurrence. In their study, 75.2% of patients undergoing PVI alone achieved AF-free survival, compared with 66.8% in the group receiving additional ablation. The authors suggested that exclusive endocardial mapping may be insufficient to identify all relevant drivers, as epicardial substrates may also contribute to focal activation [8]. Moreover, prior studies have indicated that ablation itself may facilitate the development of reentrant circuits along prior lesion lines [27]. Post-ablation scarring has also been implicated in AF recurrence, supporting the hypothesis that extensive or repetitive lesion sets may inadvertently create new arrhythmogenic substrates [28]. Similarly, Atienza et al. reported no significant advantage of combined ablation strategies over conventional circumferential PVI (CPVI) or high-frequency source ablation (HFSA) alone, while observing a higher complication rate, underscoring the need for improved mapping and ablation techniques [10].

Although freedom from AF was modestly improved, the addition of driver ablation to PVI did not result in a statistically significant improvement in freedom from ATA (RR 1.09; 95% CI, [0.98, 1.20]; *p* = 0.11). ATA is a well-recognized complication following AF ablation, frequently arising from atrial remodeling and scar-related conduction pathways. Post-ablation ATA most commonly presents as macroreentrant tachycardia, although microreentrant and focal mechanisms also play contributory roles [28, 29]. Spatial gaps within ablation lesions may give rise to areas of slow conduction and fixed anatomical boundaries, thereby creating a substrate conducive to reentrant ATAs and atrial flutter [30]. To address these challenges, advanced mapping techniques such as phase mapping, electrogram flow mapping, and three-dimensional electroanatomical mapping have been increasingly utilized to refine lesion targeting and minimize proarrhythmic substrates [29, 31].

The studies included in this meta-analysis employed a variety of adjunctive mapping techniques in addition to PVI, with most reporting longer procedure times, increased fluoroscopy exposure, and prolonged ablation durations associated with combined strategies. However, Baek et al. observed comparable procedural and ablation times between intervention groups, likely because patients undergoing PVI alone also underwent DF mapping, albeit without subsequent DF ablation [18]. These findings indicate a procedural trade-off, in which a modest increase in freedom from AF was observed at the cost of prolonged procedural and ablation durations.

Across all included studies, the incidence of procedural complications was low and comparable between groups, suggesting that adjunctive atrial driver ablation can be performed with an acceptable safety profile. Atienza et al. noted a trend toward increased complications with combined ablation, potentially attributable to incomplete driver elimination, extensive atrial fibrosis, and inherent limitations in mapping persistent AF [10]. Similarly, Deisenhofer et al. reported comparable complication rates between PVI alone and driver-guided ablation groups; although two deaths occurred in the tailored ablation group, these events were deemed unrelated to the ablation procedures [17]. Collectively, these findings suggest that while atrial driver ablation does not consistently increase overall complication rates, it is associated with longer procedures, greater radiation exposure, and a small but clinically relevant risk of severe adverse events.

### Limitations

Our study has numerous important limitations that warrant consideration. Statistically, substantial heterogeneity was observed across the included studies, likely reflecting differences in mapping technologies, ablation strategies, operator experience, and baseline patient characteristics. In addition, the approaches used to identify and ablate atrial drivers were heterogeneous, encompassing FIRM, dominant frequency mapping, and electrogram dispersion–based techniques, thereby limiting direct comparability of outcomes across trials. The relatively small number of eligible studies further constrained the pooled sample size, potentially reducing statistical power. Furthermore, the study parameters were not consistently reported across all studies, further reducing the number of studies available for each parameter. In addition, the subgroup analyses stratified by atrial driver ablation strategy were underpowered because of the limited number of studies within each subgroup. Together with the substantial between-study heterogeneity and the instability of the pooled estimates observed in sensitivity analyses, these findings suggest that the apparent benefit of atrial driver ablation should be interpreted cautiously and considered hypothesis-generating. Furthermore, one included study was assessed as having some concerns regarding risk of bias, further limiting the certainty of the available evidence. Consequently, the results of this meta-analysis should be interpreted cautiously. Nonetheless, despite these limitations, the pooled evidence provides clinically meaningful insights. Several studies highlighted the critical role of mapping methodology, patient selection, and individualized ablation strategies, which may collectively contribute to improved procedural efficacy and more consistent clinical outcomes.

## Conclusion

In conclusion, adjunctive atrial driver ablation may modestly increase the likelihood of freedom from AF in patients with PeAF and LS-PeAF, however, this benefit does not translate into a significant reduction in ATA recurrence. Nevertheless, this finding was not supported by a robust statistical model. Although the addition of atrial driver ablation is associated with longer procedural, fluoroscopy, and ablation times, its overall safety profile remains comparable to that of conventional pulmonary vein isolation. Taken together, the current evidence suggests that adjunctive atrial driver ablation strategies warrant further investigation in patients with AF, given the uncertainty surrounding the magnitude of benefit and the heterogeneity among available mapping approaches. Future studies assessing individual atrial driver mapping methodologies are needed to establish the most clinically effective and balanced approach for guiding atrial driver ablation in patients with AF. Additionally, the efficacy of atrial driver ablation should be further delineated in specific patient populations, particularly those with more advanced atrial remodeling.

## Supplementary Information

Below is the link to the electronic supplementary material.

Supplementary Material 1

## Data Availability

The authors confirm that the data supporting the findings are available within the article (and its supplementary information file).

## Abbreviations

AF: Atrial fibrillation
ATA: Any atrial tachyarrhythmia
CFAE: Complex fractionated atrial electrograms
CPVI: Circumferential pulmonary vein isolation
FIRM: Focal impulse and rotor modulation
HFSA: High-frequency source ablation
LFHS: Local high-frequency source
LS-PeAF: Long-standing persistent atrial fibrillation
PeAF: Persistent atrial fibrillation
PV: Pulmonary vein
PVI: Pulmonary vein isolation
TIA: Transient ischemic attack
