# Supplementary material for: The efficacy of additional atrial driver ablation in persistent and long-standing persistent atrial fibrillation: a systematic review and meta-analysis of randomized controlled trials
Source: Egypt Heart J. 2026 Nov 2;78:80. doi: 10.1186/s43044-026-00792-x (PMC13728090; doi:10.1186/s43044-026-00792-x)
Supplement: Supplementary file 1 [file 43044_2026_792_MOESM1_ESM.docx]

**SUPPLEMENTARY MATERIAL**

**The efficacy of additional atrial driver ablation in persistent and long-standing persistent atrial fibrillation: A systematic review and meta-analysis of randomized controlled trials**

**Supplementary Table 1.** Search Strategy

**Supplementary Figure 1.** Subgroup analyses of efficacy outcomes according to atrial driver ablation approaches

**Supplementary Table 1. Search Strategy**

| **Database** | **Keywords** | **Filters** |
| --- | --- | --- |
| **PubMed** | (((("atrial fibrillation"[Title/Abstract]) OR ("AF"[Title/Abstract])) AND (("persistent atrial fibrillation"[Title/Abstract]) OR ("long-standing persistent atrial fibrillation"[Title/Abstract]) OR ("longstanding persistent atrial fibrillation"[Title/Abstract]))) AND (("catheter ablation"[Title/Abstract]) OR ("atrial driver ablation") OR ("rotor ablation") OR ("focal impulse and rotor modulation") OR ("FIRM") OR ("driver ablation") OR ("driver mapping") OR ("mapping-guided ablation") OR ("computational mapping"))) AND (("randomized controlled trial"[Title/Abstract]) OR ("randomized"[Title/Abstract]) OR ("randomised"[Title/Abstract]) OR ("RCT"[Title/Abstract])) | Title/Abstract; Clinical Study, Clinical Trial, Multicenter Study, Observational Study, Randomized Controlled Trial |
| **Scopus** | ("atrial fibrillation") OR ("AF")  AND  ("persistent atrial fibrillation") OR ("long-standing persistent atrial fibrillation") OR ("longstanding persistent atrial fibrillation")  AND  ("catheter ablation") OR ("atrial driver ablation") OR ("rotor ablation") OR ("focal impulse and rotor modulation") OR ("FIRM") OR ("driver ablation") OR ("driver mapping") OR ("mapping-guided ablation") OR ("computational mapping")  AND  ("randomized controlled trial") OR ("randomized") OR ("randomised") OR ("RCT") | Article title, Abstract, Keywords; Article |
| **Europe PMC** | (((("atrial fibrillation") OR ("AF")) AND (("persistent atrial fibrillation") OR ("long-standing persistent atrial fibrillation") OR ("longstanding persistent atrial fibrillation"))) AND (("catheter ablation") OR ("atrial driver ablation") OR ("rotor ablation") OR ("focal impulse and rotor modulation") OR ("FIRM") OR ("driver ablation") OR ("driver mapping") OR ("mapping-guided ablation") OR ("computational mapping"))) AND (("randomized controlled trial") OR ("randomized") OR ("randomised") OR ("RCT")) AND (((SRC:MED OR SRC:PMC OR SRC:AGR OR SRC:CBA) NOT (PUB_TYPE:"Review"))) | Research Articles |
| **Cochrane Library** | ("atrial fibrillation") OR ("AF")  AND  ("persistent atrial fibrillation") OR ("long-standing persistent atrial fibrillation") OR ("longstanding persistent atrial fibrillation")  AND  ("catheter ablation") OR ("atrial driver ablation") OR ("rotor ablation") OR ("focal impulse and rotor modulation") OR ("FIRM") OR ("driver ablation") OR ("driver mapping") OR ("mapping-guided ablation") OR ("computational mapping")  AND  ("randomized controlled trial") OR ("randomized") OR ("randomised") OR ("RCT") | Title, Abstract, Keywords |

**Supplementary Figure 1.** Subgroup analyses of efficacy outcomes according to atrial driver ablation approaches

1. Freedom from AF

2. Freedom from Any ATA
